# Neprilysin Inhibitor–Angiotensin II Receptor Blocker Combination Therapy (Sacubitril/valsartan) Suppresses Atherosclerotic Plaque Formation and Inhibits Inflammation in Apolipoprotein E- Deficient Mice

**DOI:** 10.1038/s41598-019-42994-1

**Published:** 2019-04-24

**Authors:** Hui Zhang, Gangqiong Liu, Wenping Zhou, Wenjing Zhang, Kai Wang, Jinying Zhang

**Affiliations:** 1 Department of Cardiology, the First Affiliated Hospital of Zhengzhou University, Zhengzhou, Henan P.R. China; 2 Department of Cardiology, the Third Affiliated Hospital of Zhengzhou University, Zhengzhou, Henan P.R. China

**Keywords:** Cytokines, Drug discovery

## Abstract

We assessed the effects of the sacubitril/valsartan combination drug (LCZ696), in comparison to valsartan alone, on the progression of atherosclerotic plaque formation and inflammatory gene expression in apolipoprotein E- deficient mice (apoE^−/−^ mice). Seventy-two apoE^−/−^ mice were fed a western diet and a constrictive silastic tube was used to elicit carotid lesion formation. The animals were separated into a control group, a valsartan group or an LCZ696 group (n = 24 in each group). Plaques in the carotid artery were harvested 12 weeks later for histological examination. The levels of pro-inflammatory genes in the plasma and lesions were detected using real-time PCR and ELISA. Valsartan or LCZ696 treatment remarkably inhibited the expression of pro-inflammatory genes, including interleukin-6, matrix metalloproteinase-8 and monocyte chemotactic protein-1, in comparison with the control group. Meanwhile, both valsartan and LCZ696 suppressed the formation of atherosclerotic plaques by decreasing plaque lipid content and cross-sectional plaque area and increasing the content of plaque collagen and fibrous cap thickness. In particular, LCZ696 performed the best in suppressing atherosclerosis and inhibiting the level of pro-inflammatory genes. LCZ696 significantly ameliorated atherosclerosis and inflammation in apoE^−/−^ mice compared with valsartan.

## Introduction

Atherosclerosis, a multi-factorial disease intertwined with inflammation, accounts for the majority of morbidity and mortality worldwide^1^. The renin angiotensin system (RAS) is tightly connected with the pathophysiology of atherosclerosis and its clinical complications^2^. Clinical and experimental investigations have shown that the angiotensin II receptor blocker (ARB) valsartan inhibits the RAS, exerts beneficial effects on plaque stability and reduces acute coronary events by suppressing inflammatory cytokines^2,3^. ARBs have been demonstrated to be the cornerstone for the treatment of atherosclerosis and heart failure (HF)^4–6^.

The dual neprilysin inhibitor, sacubitril, and angiotensin II (ang II) receptor blocker, valsartan, combined drug (sacubitril/valsartan) is a novel cardiovascular drug that consists of molecular moieties of the neprilysin inhibitor and the ARB in a 1:1 ratio, the so-called angiotensin receptor neprilysin inhibitor (ARNI) LCZ696^7^. LCZ696 has been proven to be more effective than classical renin**–**angiotensin system blockers (including ARBs and angiotensin converting enzyme inhibitors) for the treatment of congestive HF^4,8,9^. Sacubitril/valsartan simultaneously suppresses the angiotensin II receptor and neprilysin, and exerts beneficial effects on endothelial dysfunction, cardiac dysfunction, hypertension, HF, ischemic brain damage and cardiovascular ischemia–reperfusion injury in experimental and clinical investigations^4,9^.

However, whether the ARNI LCZ696 has similar anti-atherogenic effects remains controversial. It is unclear whether LCZ696 can lead to amelioration of established atherosclerotic plaques in mouse models. The objective of the current work was to determine the impacts of the ARNI LCZ696 on the progression of carotid lesions and pro-inflammatory cytokines in collar-elicited atherosclerotic plaques in apolipoprotein E-deficient mice (apoE^−/−^ mice). Further, our study clarifies whether LCZ696 has anti-atherogenic and anti-inflammatory effects beyond the RAS blockade by comparison with the traditional ARB valsartan.

## Results

### Valsartan/LBQ657 and valsartan suppressed the levels of pro-inflammatory cytokines *in vitro*

**Figure 1:**
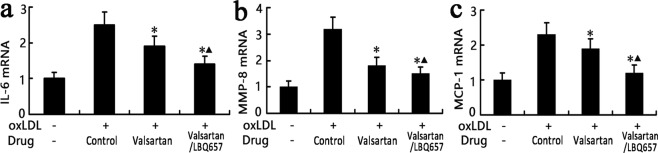
Effects of valsartan and valsartan/LBQ657 *in vitro*. (**a**–**c**) qRT PCR analysis of IL-6, MMP-8 and MCP-1 mRNA levels in RAW 264.7 cells. After incubation with 60 μg/mL oxLDL, the mRNA levels of IL-6, MMP-8 and MCP-1 increased sharply. Valsartan/LBQ657 or valsartan inhibited the augmentation of IL-6 (**a**), MMP-8 (**b**) and MCP-1 (**c**) induced by oxLDL. Valsartan/LBQ657 was superior to valsartan alone in normalizing these inflammatory markers. Symbols + and −denote the presence and absence of valsartan/LBQ657 or oxLDL. *P < 0.05 vs. control group; ^▲^*P* < 0.05 vs. valsartan group. MMP-8: matrix metalloproteinase-8; IL-6: interleukin-6; MCP-1: monocyte chemotactic protein-1; oxLDL: oxygenized low density lipoprotein.

Before oxygenized low-density lipoprotein (oxLDL) pretreatment, RAW264.7 cells had low levels of pro-inflammatory cytokine expression, including interleukin-6 (IL-6), matrix metalloproteinase-8 (MMP-8), and monocyte chemotactic protein-1 (MCP-1). After stimulation with oxLDL (60 μg/mL), the mRNA levels of these pro-inflammatory cytokines increased sharply. We used valsartan/LBQ657 instead of LCZ696 in our *in vitro* investigation because LCZ696 did not separate into valsartan and sacubitril in cell-based experiments. LBQ657 is an active form of sacubitril. Our results demonstrated that valsartan/LBQ657 or valsartan suppressed the high expression of IL-6, MMP-8 and MCP-1 provoked by oxLDL *in vitro* (*P* < 0.05, Fig. 1). In addition, valsartan/LBQ657 was superior to valsartan in normalizing these inflammatory markers (*P* < 0.05, Fig. 1).

### LCZ696 and valsartan had no significant effects on plasma lipid indicators or body weight

The mean body weights of the animals in the control group (28.91 ± 3.42 g), valsartan group (29.25 ± 3.68 g) and LCZ696 group (28.66 ± 3.53 g) were similar, indicating that LCZ696 or valsartan administration did not affect body weight. Likewise, we found no differences with respect to plasma triglyceride (TG) and total cholesterol (TC) concentrations among the control group (31.35 ± 2.95 and 2.85 ± 0.63 mmol/liter), the valsartan group (32.13 ± 3.16 and 2.91 ± 0.72 mmol/liter) and the LCZ696 group (31.78 ± 3.87 and 2.84 ± 0.68 mmol/liter). These results revealed that neither valsartan nor LCZ696 treatment altered plasma lipid indicators. Both valsartan and LCZ696 were tolerated very well in the present study and the mice remained in good health throughout the experiments.

### LCZ696 and valsartan altered plasma concentrations of aldosterone and BNP

**Figure 2:**
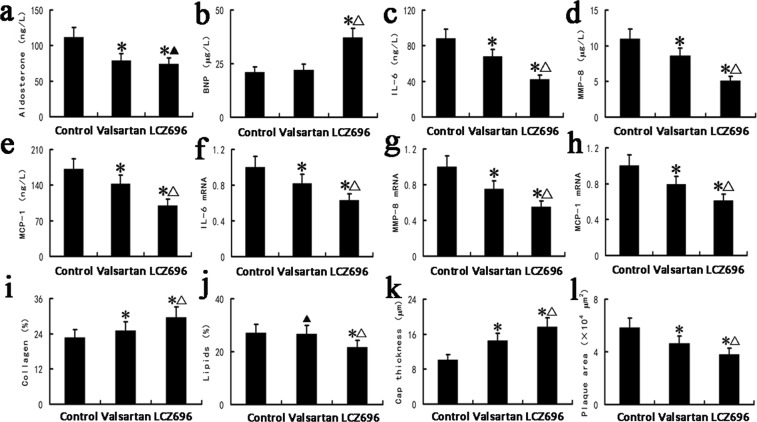
(**a**–**e**) Show the plasma concentration of aldosterone (**a**), BNP (**b**), IL-6 (**c**), MMP-8 (**d**) and MCP-1 (**e**) in the control, valsartan and LCZ696 groups (n = 24). (**f**–**h**) Show the carotid plaque mRNA levels of IL-6 (**f**), MMP-8 (**g**) and MCP-1 (**h**) in the control, valsartan and LCZ696 groups. (**i**–**l**) Are quantification of atherosclerotic lesion collagen content (**i**), lipid content (**j**), fibrous cap thickness (**k**) and plaque area (**l**) in the control, valsartan and LCZ696 groups. *P < 0.05 vs. control group; ^Δ^*P* < 0.05 vs. valsartan group; ^▲^*P* > 0.05 vs. valsartan group (n = 24).

In the current study, we evaluated the concentration of aldosterone, the end product of the RAS, and the concentration of BNP, the end product of the natriuretic peptide (NP) system in the plasma. Our results revealed that valsartan or LCZ696 administration remarkably decreased plasma concentration of aldosterone in these animals (Fig. 2a, *P* < 0.05). The plasma aldosterone levels of the valsartan group and the LCZ696 group were comparable (Fig. 2a, *P* > 0.05). Meanwhile, LCZ696 treatment notably increased plasma BNP concentration in these animals (Fig. 2b, *P* < 0.05). The plasma BNP levels of the valsartan group and the control group were similar (Fig. 2b, *P* > 0.05).

### LCZ696 or valsartan treatment attenuated the concentrations of pro-inflammatory cytokines in the plasma

Both LCZ696 and valsartan treatment attenuated the concentration of pro-inflammatory cytokines, including MMP-8, IL-6 and MCP-1 in the plasma (all *P* < 0.05). LCZ696 performed the best in inhibiting these pro-inflammatory cytokines (Fig. 2c–e, all *P* < 0.01).

### LCZ696 and valsartan altered the relative distribution of white blood cells (WBCs) in apoE^−/−^ mice

**Figure 3:**
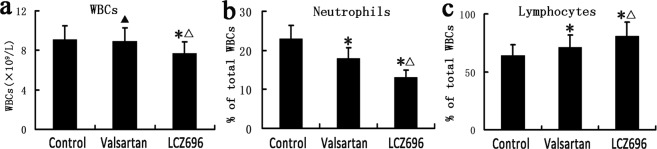
Altered specific circulating WBC levels. (**a**–**c**) Show WBCs (**a**), relative neutrophil (**b**) and lymphocyte (**c**) counts in the control, valsartan and LCZ696 groups. *P < 0.05 vs. control group; ^Δ^*P* < 0.05 vs. valsartan group; ^▲^*P* > 0.05 vs. control group (n = 24). WBCs: white blood cells.

Valsartan administration remarkably reduced the number of circulating neutrophils, and enhanced circulating lymphocytes in these animals in comparison with the control group. Moreover, LCZ696 administration exerted more beneficial effects than valsartan treatment in decreasing the total cell counts of circulating WBCs and neutrophils, and increasing the cell counts of circulating lymphocytes (Fig. 3, *P* < 0.05). In addition, compared with the control group, we found a decreasing tendency in the total cell count of circulating WBCs under valsartan treatment, although the results were not statistically significant (Fig. 3a, *P* > 0.05). Our data are in accordance with the amelioration of the systemic inflammatory response in the two treatment groups. LCZ696 performed the best in inhibiting systemic inflammation (Fig. 3, *P* < 0.05).

### LCZ696 and valsartan suppressed the formation of atherosclerotic plaques

**Figure 4:**
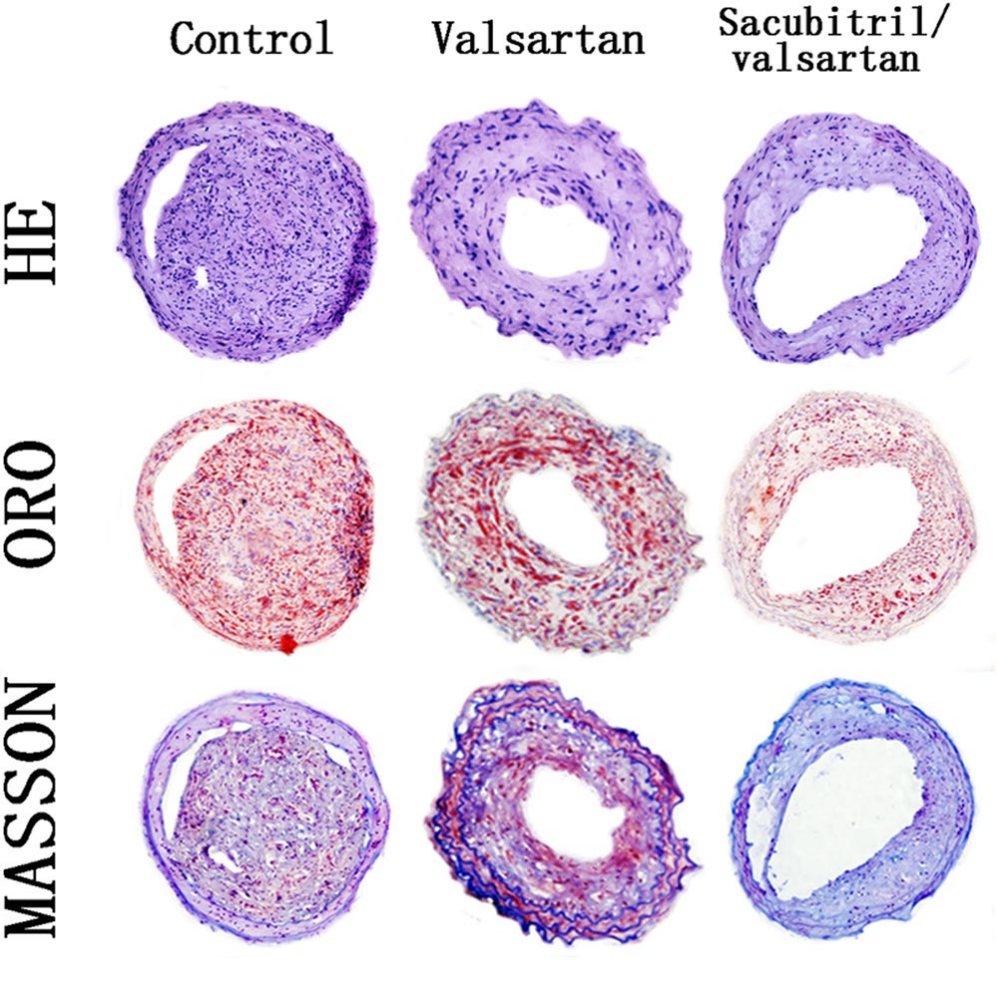
Histological staining showing plaque composition in the control, valsartan and LCZ696 groups. Representative images were stained with H&E, ORO and Masson’s trichrome; magnification 200×.

As shown in Fig. 4 and Fig. 2i, valsartan treatment increased the collagen content of plaques compared with the control group, while the LCZ696 group exhibited the highest collagen content (Figs 2i and 4, P < 0.05).

Compared with the mice treated with valsartan, LCZ696 treatment notably decreased the severity of lipid deposition in these animals (Figs 2j and 4, *P* < 0.05). In addition, compared with the animals in the control group, we found a decreasing trend in the plaque lipid content of the mice treated with valsartan, although the difference was not statistically significant (Figs 2j and 4, *P* > 0.05).

As shown in Figs 4 and Fig. 2k, valsartan treatment remarkably increased the fibrous cap thickness in the carotid lesions of the animals in comparison with animals in the control group. The fibrous cap thickness was greatest in the mice treated with LCZ696 (Figs 2k and 4, *P* < 0.05).

The mice in the valsartan group were found to have a smaller plaque area in comparison with the animals in the control group. In addition, the plaque area was further reduced in the LCZ696-treated mice compared to the mice receiving valsartan treatment (Figs 2l and 4, *P* < 0.05).

Although the two treatments were both effective in suppressing atherosclerosis, the LCZ696 group displayed enhanced amelioration of atherosclerosis and plaque stability, in comparison with the valsartan group (Figs 2 and 4).

### LCZ696 and valsartan inhibited the expression of inflammatory genes within carotid plaques

The mRNA levels of inflammatory genes in the carotid lesions of the animals, including MMP-8, IL-6 and MCP-1, reduced strikingly following LCZ696 or valsartan treatment (all *P* < 0.01). In addition, LCZ696 was found to be more effective than valsartan in inhibiting the mRNA level of all three of these inflammatory genes in the lesions (Fig. 2f–h, all *P* < 0.01).

## Discussion

In the current investigation, we applied the collar-elicited atherosclerosis mouse model to examine how the ARNI LCZ696 regulates the process of atherosclerosis and the associated inflammatory reaction. Furthermore, we compared the effects of this ARNI with the traditional ARB valsartan, which has been demonstrated to be effective on established lesions in apoE^−/−^ mice^5,8,10^. We demonstrated that LCZ696 or valsartan treatment attenuated the expressions of pro-inflammatory cytokines, including IL-6, MMP-8 and MCP-1, suppressed atherosclerosis and increased plaque stability. In addition, the dual ARB/neprilysin inhibitor LCZ696 was found to be more effective than valsartan alone in ameliorating inflammation and atherosclerosis and increasing plaque stability.

Atherosclerotic plaque formation is a complex inflammatory disease of the arterial wall^11–13^. The RAS is involved in all stages of atherosclerosis^2^. The RAS has pro-atherogenic, pro-inflammatory and pro-fibrotic effects beyond its primary role in adjusting arterial pressure. Blocking the RAS exerts beneficial effects for the management of atherosclerosis and HF. The regression of atherosclerosis in animal models by blocking the RAS with an ARB is well documented^2,4,10^. ARBs have been demonstrated to be the cornerstone for the management of atherosclerosis and HF^4–6^. The dual neprilysin inhibitor sacubitril and ang II type 1 receptor blocker valsartan combined drug sacubitril/valsartan (LCZ696) suppresses the neprilysin and ang II receptor at the same time and exerts beneficial effects on endothelial dysfunction, cardiac dysfunction, hypertension, HF, ischemic brain damage and cardiovascular ischemia–reperfusion injury in experimental and clinical investigations^4,7,9^. Neprilysin causes the decomposition reaction of many vasoactive peptides, such as natriuretic peptides, adrenomedullin, substance P and bradykinin^4,5,7^. Suppressing the bioactivity of neprilysin enhanced the concentrations of these vasoactive peptides, inhibiting the neurohormonal overactivation of ang II and aldosterone that results in vasoconstriction, sympathetic activation, sodium retention, and atherosclerotic plaque formation^4,5,7^. Apart from its major function in inhibiting the ang II type 1 receptor, the ARNI LCZ696 reduces natriuretic peptide degradation by suppressing neprilysin. Natriuretic peptides increase urinary sodium excretion, act as vasodilators, suppress aldosterone generation and inhibit the activity of the sympathetic system^9,14^. Previous studies have demonstrated that LCZ696 (sacubitril/valsartan) is superior to traditional RAS blockers in patients with congestive HF^4,5,9,10^. However, whether LCZ696 has similar anti-atherogenic effects to valsartan remains controversial. We found evidence that LCZ696 exerts anti-atherogenic effects in clinical and experimental studies: in the PARADIGM-HF study and the PARAMOUNT investigation, compared with traditional RAS blockers, LCZ696 treatment not only reduced the primary endpoint but also decreased the coronary composite outcome in patients with HF after myocardial infarction, suggesting that LCZ696 has anti-atherogenic effects beyond HF^5,15^. In addition, LCZ696 suppressed the angiotensin II receptor and neprilysin simultaneously and improved endothelial dysfunction, cardiac dysfunction, sympathetic activation, sodium retention and ischemia–reperfusion injury in animal and clinical studies, suggesting a possible anti-atherogenic role of LCZ696^4,9^. ARBs have been demonstrated to be efficacious in ameliorating atherosclerosis in animal models^2,4,10^. However, it remains unclear whether the ARNI LCZ696 can suppress established atherosclerotic plaques in mouse models. Thus, we explored the functions of the ARNI LCZ696, compared with the traditional RAS blocker valsartan, on the atherosclerotic plaque and inflammation process in atherosclerosis-prone apoE^−/−^ mice.

The key finding of our present work is that the levels of proinflammatory cytokines in the plaques and plasma of the animals can be effectively down-regulated following LCZ696 or valsartan treatment, leading to reduced plaque area and plaque content of lipid, and enhanced content of collagen and fibrous cap thickness in carotid lesions in mice, indicating improved stability. More importantly, the dual ARB/neprilysin inhibitor LCZ696 ameliorated atherosclerosis and inflammation more efficiently than the ARB valsartan alone. Thus, in view of these results, it is reasonable to speculate that the positive effects of LCZ696 treatment may be the result of combined ang II type 1 receptor blockade and neprilysin suppression rather than RAS blockade alone. Possible explanations for this therapeutic reaction may be that LCZ696 reduced the levels of proinflammatory genes and attenuated the accumulation of macrophages in the lesion. Meanwhile, *in vitro*, simultaneous administration of the two vasoactive systems (i.e., suppression of the RAS by the ARB valsartan and augmentation of the NP system by the neprilysin inhibitor sacubitril) may attenuate the levels of MMP-8, IL-6 and MCP-1 more efficiently than RAS blockade by valsartan alone in RAW264.7 cells. The proinflammatory cytokines IL-6, MMP-8, MCP-1 and ang II are responsible for the recruitment of macrophages to the vessel wall, where they promote the generation of more cytokines and chemokines and perpetuate the inflammatory response at the injury site^16^. Macrophages are the major source of MMP-8, MCP-1 and IL-6 in atherosclerotic plaques. By virtue of these reactions, macrophages, ang II and these pro-inflammatory cytokines contribute to a positive-regeneration circle of inflammatory reaction and atherosclerotic plaque formation. These cytokines and chemokines are involved in vessel inflammation, vulnerable plaque rupture and thrombosis. Therefore, LCZ696 might regulate the process of anti-atherosclerosis and anti-inflammation. In addition, the LCZ696 group had lower circulating WBCs and neutrophils, and higher circulating lymphocytes, than the valsartan and control groups, which may serve as indicators to predict the stability of the lesions^17^. Our current findings are in line with the amelioration of systemic inflammation in animals receiving LCZ696 treatment, suggesting that LCZ696 can exert a potentially anti-inflammatory and anti-atherogenic role.

The RAS and the NP system each serve as a negative feedback inhibitor of the activity of the other^9^. We mainly found that LCZ696 or valsartan can effectively down-regulate the activity of the RAS and up-regulate the activity of the NP system. In the current investigation, the plasma concentration of aldosterone, the end product of the RAS, was remarkably lower in the mice receiving LCZ696 or valsartan treatment in comparison with the control group. In addition, the plasma level of BNP, the end product of the NP system, was notably elevated in the mice receiving LCZ696 treatment in comparison with the control and valsartan groups, suggesting that the equilibrium between the RAS and the NP system may favor an anti-atherogenic role in the LCZ696 group.

In the present investigation, both valsartan and LCZ696 stabilized plaques by decreasing their lipid content and cross-sectional area and increasing their content of collagen and fibrous cap thickness. Of the treatments tested, LCZ696 performed the best, supporting the idea that dual neprilysin and ang II receptor suppression with LCZ696 was superior to ang II type 1 receptor blockade with valsartan alone in stabilizing plaques. The sound effects of RAS inhibition against atherosclerotic lesion formation are potentially increased by the augmentation in NP level as a result of suppression of NP degradation^9^. Neprilysin contributes to the degradation of many endogenous vasoactive peptides, such as natriuretic peptides and bradykinin^4,5,7,18,19^. These endogenous vasoactive peptides inhibit the harmful cardiovascular reactions of ang II and aldosterone in patients with atherosclerosis and HF, such as endothelial dysfunction, sympathetic activation, vasoconstriction, hypertrophy and sodium retention^18–21^. In addition, BNP has been demonstrated to ameliorate ang II-stimulated neurohormonal overactivation, aldosterone synthesis, endothelial dysfunction and atherogenesis^18,19^. In the current work, the plaques in the control group displayed relatively higher levels of proinflammatory cytokines, such as IL-6, MMP-8 and MCP-1, and this result was notably reversed by LCZ696 or valsartan administration. More importantly, dual ang II receptor and neprilysin inhibition with LCZ696 reduced expression of these inflammatory genes more effectively than ang II receptor blockade with valsartan alone.

We did not include a sacubitril alone group in the present study because a selective neprilysin blocker, such as sacubitril or candoxatril, enhanced not only the plasma concentrations of BNP, but also those of ang II and thus abolished the effects of the former^22^.

Limitations of this study should be considered. Firstly, neprilysin leads to the decomposition reaction of numerous vasoactive peptides. Apart from its main role in degrading NPs, neprilysin also cleaves other endogenous vasoactive peptides such as bradykinin, adrenomedullin, substance P and calcitonin gene-related peptide^22,23^. Thus, it is logical to infer that the greater beneficial effects of LCZ696 compared to valsartan in apoE^−/−^ mice might be partially attributed to other peptides than NPs. Secondly, the present study was undertaken in apoE^−/−^ mice without HF. However, patients with HF usually have a very high concentration of BNP in the plasma. Consequently, care should be taken when generalizing these data to patients with HF. Additionally, the collar-elicited animal model of atherosclerosis does not completely mimic the progression of atherosclerotic plaque rupture and intraluminal thrombosis in humans^4,18,24–26^. Finally, compared with the control group, we found a decreasing tendency in the plaque lipid content of the mice when treated with valsartan, although this was not statistically significant. One possible reason for this phenomenon might be that the doses of valsartan and LCZ696 used in the current work were relatively low^18^. We cannot rule out the possibility that a higher dose of valsartan and LCZ696 would have a profound effect on lipid content and inflammatory processes in the plaques of the mice^8,9,18^. We will further explore this issue in the near future.

## Conclusion

In summary, dual neprilysin inhibitor–angiotensin II receptor blocker combination therapy with LCZ696 was superior to ang II receptor blockade with valsartan alone in suppressing atherosclerosis and inhibiting the inflammatory response in apoE^−/−^ mice. The effects of LCZ696 or valsartan were independent of plasma lipoprotein level. Our current work demonstrates that LCZ696 may provide a novel therapeutic method to treat atherosclerosis in the future.

## Methods

### Cell culture

The mouse macrophage cell line RAW264.7 was maintained in DMEM in a 5% CO_2_ and 95% air humidified atmosphere. The levels of IL-6, MMP-8 and MCP-1 in mouse macrophage cells are very low, so we incubated the RAW264.7 cells with 60 µg/mL of oxygenized low density lipoprotein (oxLDL). Then, the effects of valsartan/LBQ657 and valsartan alone on the levels of these pro-inflammatory cytokines were analyzed by qRT-PCR.

### Animal protocol

Seventy-two male apoE^−/−^ mice (12 weeks old) with the C57BL/6 background were obtained from Beijing University. The animals were fed a western diet in the present study. The western diet, containing 15% cocoa butter and 0.25% cholesterol, was obtained from the Animal Center of Zhengzhou University. The body weight of the control (23.46 ± 3.08 g), valsartan (23.79 ± 3.32 g) and LCZ696 (23.55 ± 3.28 g) groups were comparable. After anesthesia (40 mg/kg pentobarbital sodium i.p.) a constrictive silastic tube (0.30 mm) was applied to elicit plaque formation^1,11,12^. The mice were divided into three groups (n = 24 in each group): the control group, the valsartan group and the LCZ696 group. At week 8, the collars around the carotid artery were removed, and the mice were given 3 mg/kg valsartan (the valsartan group) or 6 mg/kg LCZ696 (the LCZ696 group) dissolved in corn oil, or corn oil alone (the control group) every day for 12 weeks by oral gavage. The LCZ696 group and the valsartan group were named the treatment groups in the present study. The animals were sacrificed at week 20. The housing and care of animals and all of the procedures performed in the present study complied with the Animal Management Rules of the Chinese Ministry of Health (Document No. 55, 2001) and were approved by the Ethics Committee of Zhengzhou University (Zhengzhou, China).

### Histological analyses of plaques

At week 20, animals were sacrificed by intraperitoneal injection with pentobarbital sodium for histological examination of plaques. The carotid arteries of the mice were perfused with PBS and 4% formaldehyde via the left ventricle. Then the plaques in the carotid artery were dissected and embedded in optimal cutting temperature (OCT) compound and cut into 5 μm sections using a Leica freezing microtome (5 μm sections at 50 μm intervals). The lesions from each animal were serially sectioned and stained with hematoxylin and eosin (HE), Masson’s trichrome and oil red O (ORO). Images of the plaques were captured using an Olympus microscope (Olympus, Tokyo, Japan). The cross-sectional area of the plaque in each section was determined using automated computer-assisted morphometry. The percentages of positively-stained collagen and lipid areas were calculated by computer-assisted morphometry^13^.

### Plasma lipid and biological examination

Blood samples were collected from the retro-orbital plexus after an overnight fast, and plasma was collected and centrifuged at 1,500 × *g* for 15 min. Next, plasma was separated to detect the concentrations of aldosterone, brain natriuretic peptide (BNP), IL-6, MMP-8, MCP-1, TG and TC using commercial kits (CoWin Bioscience, Beijing, China).

### Complete blood cell count

Blood samples were collected into EDTA-coated tubes. A complete blood cell count was performed using a UniCel DxH 800 hematology analyzer (Beckman Coulter, Brea, CA, USA) in accordance with the supplier’s protocols.

### Real-time quantitative PCR (qPCR) analysis

Total RNA from the cells or the carotid arteries was extracted using Trizol reagent in accordance with the supplier’s instructions (CoWin Bioscience). qPCR analysis was conducted as described previously^1,11,12^. The relative expressions of IL-6, MCP-1 and MMP-8 mRNAs were quantified using SYBR green with the ABI Prism 7500 sequence detection system. The forward and reverse primers were: *MCP-1*, 5′-GCTCAGCCAGATGCAGTTAACG-3′ and 5′-TCTTGGGGTCAGCACAGACCTC-3′; *MMP-8*, 5′-GCCTGACTCTGGTGATTTCTTG-3′ and 5′-TGTTGATGTCTGCTTCTCCCTG-3′; *IL-6*, 5′-ACAACCACGGCCTTCCCTACTT-3′ and 5′-TTTCTCATTTCCACGATTTCCC-3′; *β-actin*, 5′-GCTATGCTCTCCCTCACGCCAT-3′ and 5′-TCACGCACGATTTCCCTCTCAG-3′.

### Statistical analysis

All analyses were performed using SPSS Version 16.0 for Windows. Quantitative values are expressed as mean values ± standard deviation. Data were compared among groups using one-way analysis of variance (ANOVA) followed by the Student-Newman-Keuls test for post-hoc comparisons. Differences were considered to be significant if the *P*-values were less than 0.05.

## Data Availability

Data used in the present study can be obtained by contacting Hui Zhang via e-mail 55148008@qq.com.
